# In North America, Some Ovarian Cancers Express the Oncogenes of Preventable Human Papillomavirus HPV-18

**DOI:** 10.1038/srep08645

**Published:** 2015-02-27

**Authors:** Patrick Roos, Paul A. Orlando, Richard M. Fagerstrom, John W. Pepper

**Affiliations:** 1 Biometry Research Group, Division of Cancer Prevention, National Cancer Institute, 9609 Medical Center Dr., Rockville, MD. 20850; 2 Indiana University, Department of Biology, 1001 E 3rd St. Bloomington, IN, 47405; 3 Santa Fe Institute, 1399 Hyde Park Road, Santa Fe, New Mexico 87501

## Abstract

Some researchers in other regions have recommended human papillomavirus (HPV) vaccination to reduce risk of ovarian cancer, but not in North America, where evidence has previously suggested no role for HPV in ovarian cancer. Here we use a large sample of ovarian cancer transcriptomes (RNA-Seq) from The Cancer Genome Atlas (TCGA) database to address whether HPV is involved with ovarian cancer in North America. We estimate that a known high-risk type of HPV (type 18) is present and active in 1.5% of cases of ovarian epithelial cancers in the US and Canada. Our detection methods were verified by negative and positive controls, and our sequence matches indicated high validity, leading to strong confidence in our conclusions. Our results indicate that previous reports of zero prevalence of HPV in North American cases of ovarian cancer should not be considered conclusive. This is important because currently used vaccines protect against the HPV-18 that is active in ovarian tumors and, therefore, may reduce risk in North America of cancers of the ovaries as well as of the cervix and several other organ sites.

Ovarian cancer is the most lethal gynecological malignancy, with mortality within five years exceeding 50%^1^,^2^. Multiple studies have reported evidence for involvement of HPV with this cancer, usually in the form of HPV DNA in tumor cells.

Most ovarian cancers are epithelial in origin, as with HPV-caused cervical, anal, urogenital, and head-and-neck cancers. It is known that HPV does reach the ovarian epithelium, and is sometimes integrated into the genomes of epithelial ovarian cancers^3^. Therefore, some researchers have recommended HPV vaccination to reduce risk of ovarian cancer^4^, but not to our knowledge in North America, where there has been less evidence of HPV involvement in ovarian cancer.

A recent meta-analysis^3^ estimated that worldwide, 15.5% of ovarian cancers were positive for HPV DNA. By geographical region, however, DNA prevalence varied substantially, from the highest levels in Asia, to the lowest level of zero in North America. Another meta-analysis^5^ estimated a prevalence of 9% in North America, reportedly based on 8 studies comprising 211 total cases of ovarian cancer. However, none of the publications cited therein actually included data on HPV DNA prevalence in ovarian tumors in North America.

Conflicting reports of HPV DNA in ovarian cancers highlight a need both for samples from more patients and for additional detection methods. Here we report the results of a bioinformatics analysis performed on a large cohort (405 cases) based on a different detection method from those of the aforementioned meta-analyses -- using existing RNA sequence data of tumor transcriptomes to search for alignments of cDNA with viral reference sequences.

A large set of clinical samples of ovarian cancers was collected and analyzed by The Cancer Genome Atlas Research Network^6^. The resulting sequence data, including RNA sequences, was subsequently made available to other researchers. This initial effort to develop “a catalogue of molecular aberrations that cause ovarian cancer” was limited to somatic mutations of human genes, and did not investigate whether clinical samples included viral genes. Here we analyze that database in a focused effort to evaluate expression of HPV oncogenes.

In cervical cancers, HPV gene expression drives oncogenesis. Specifically, the E6 and E7 proteins of high-risk HPV types are involved in cellular transformation and are consistently expressed in cervical cancers^7^,^8^. It has also been reported that viral genome integration results in elevated expression of *E6* in cervical carcinoma^9^. Thus, in terms of carcinogenesis, evidence of HPV oncogene expression is more informative than the mere presence of HPV genes, as was assayed in most previous studies. In this study, we computationally search ovarian tumor transcriptomes for evidence of oncogene expression.

## Results

Table 1 reports the total number of samples, and the number that were HPV-positive, for each tumor type we examined.

### Validation of Methods

Our positive and negative controls supported the validity of our protocol. As expected, almost all (245 of 251 = 98%) of the cervical tumor samples produced alignments to oncogenes of at least one of the 12 high-risk HPV types. No alignments to low-risk types 6 or 11 were found.

Among those cervical tumor samples that tested positive for high-risk HPV oncogene transcripts, we detected 12 HPV types, all of which are considered high-risk types (Table 2).

We used glioblastoma tumor samples as a negative control for HPV RNA. Zero of 156 samples yielded alignments to any of the 14 HPV types we searched for. Thus, we found no evidence of any HPV type in the glioblastoma samples we tested. This also was as expected, because viruses do not normally cross the intact blood-brain barrier.

Cervical tumors most often produced alignments to HPV-16. HPV-18 was the second most prevalent type, although our method was not able to reliably distinguish between HPV-18 and HPV-45 in cervical cancers, as most samples that tested positive for one of them, also tested positive for the other. This may be because they are phylogenetically closely related^10^, and thus have high sequence similarity. All the other HPV types were rare (<10% of patients for each, Table 2). Our observed frequency of HPV in cervical samples, and the distribution of types (Table 2), agreed well with previously published literature^11^, supporting the validity of our informatics methods.

As expected, HPV-positive cervical tumors showed higher expression of viral oncogenes than did HPV-positive associated normal tissue, but with only 3 cases for which data from both cancer and normal samples were available, the difference did not reach statistical significance (Wilcoxon signed-rank test, p = 0.18).

### HPV Oncogene Expression in Ovarian Tumors

Among the ovarian cancer samples we analyzed, 6 of 405 were positive for HPV oncogene expression, with at least one read aligning to the *E6* or *E7* genes. Four of these had concordantly aligned paired reads. Six of 405 cases constituted 1.5% of our ovarian cancer cohort (the 95% confidence interval on prevalence was from 0.6% to 3.3%). Among these HPV-positive ovarian cancer samples, all reads aligning to any HPV gene were to type HPV-18. Consequently, the non-zero prevalence of HPV-18 was significantly higher than the zero prevalence of HPV-16 (McNemar's test, p = 0.031). In contrast to one isolated previous report of its genomic integration in ovarian cancer^12^, we found no evidence in ovarian tumors of HPV-6, which is considered a low-risk type. The six cases positive for HPV-18 were geographically dispersed within North America (Table 3).

For the six positive ovarian cancer samples, the number of reads aligning to HPV-18 oncogenes *E6* or *E7* were 17, 12, 6, 4, 1, and 1. In these samples, we also found reads aligning to HPV-18 genes *E1* and *E2*. Including all these genes, the six positive samples had totals of 63, 47, 16, 8, 2, and 1 read(s) aligning to the HPV-18 genome. Regarding concordancy of the paired end reads, among the six positive samples, there were 17 concordant alignments for HPV-18 oncogenes, including 12 for oncogene *E6*, and five for oncogene *E7*.

Although the prevalence of HPV transcripts in our data from ovarian tumors was not zero, with our sample sizes, its difference from prevalence in glioblastoma tumors did not reach statistical significance (Fisher's exact test, p = 0.19).

**Figure 1:**
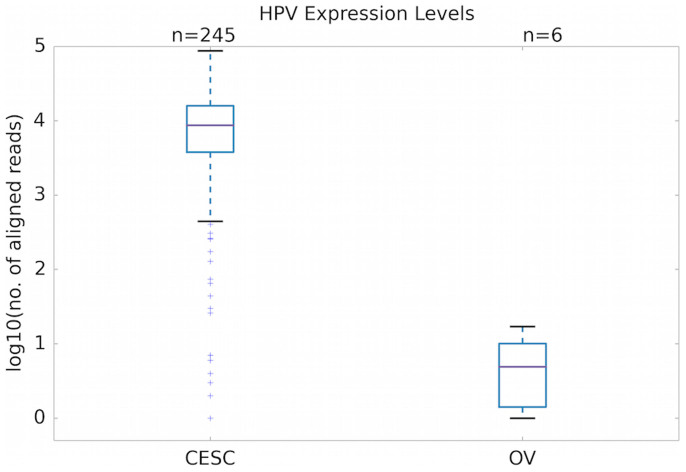
Comparison of oncogene expression, summed over all HPV types, in cervical (CESC) versus ovarian (OV) tumors, plotted on log-10 scale. Counts of aligned reads are pooled across genes *E6* and *E7*. The difference is highly significant (Wilcoxon rank-sum test, p < 10^−4^).

**Figure 2:**
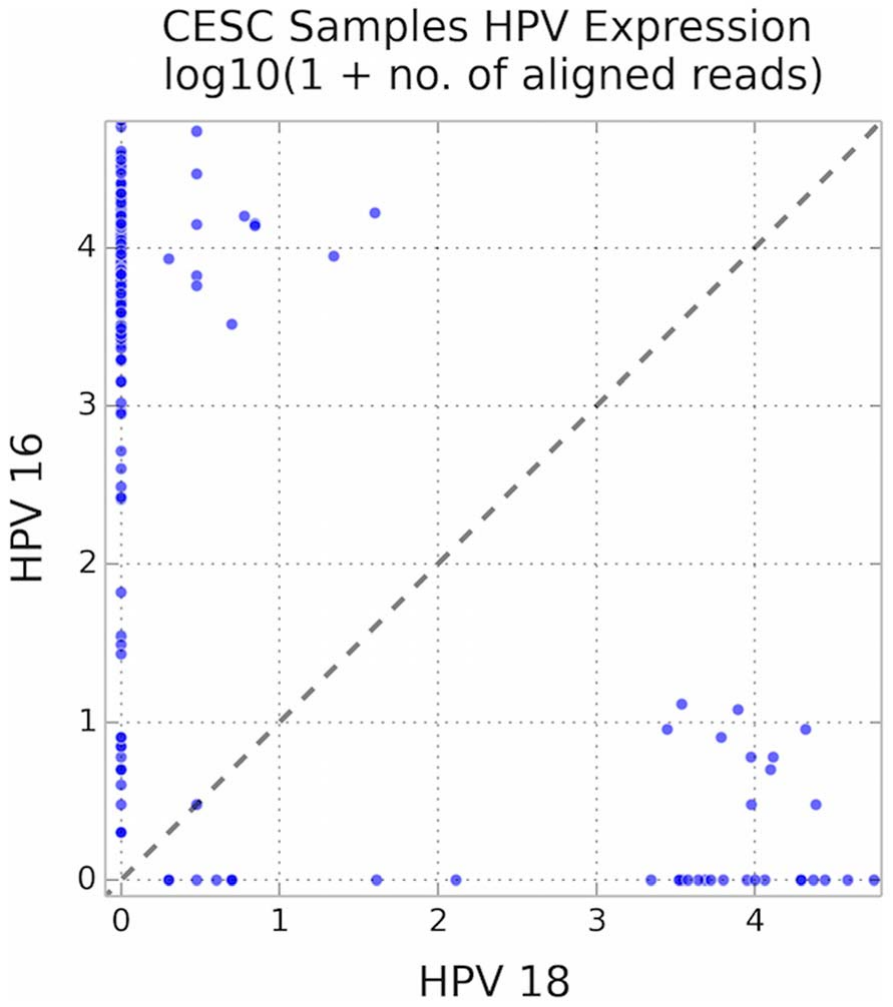
Lower expression of HPV-18 than HPV-16 within cervical cancer samples, with number of mRNA reads plotted on log-10 scale. Each dot represents one cervical tumor case. The difference in expression levels is highly significant (sign test, p < 10^−18^).

We could not determine whether ovarian tumor samples had elevated expression of HPV oncogenes relative to associated normal tissues, because RNA sequence data were not available from those normal tissues. We could, however, determine that total oncogene expression was lower in HPV-positive ovarian tumors than in HPV-positive cervical tumors (Fig. 1) (Wilcoxon rank-sum test: U = 1442.5, *p* < 10^−4^). This may not be due to any difference between the epithelial cells of the two organs, but rather to the nature of the infecting virus, which was primarily HPV-16 in cervical tumors versus exclusively HPV-18 in ovarian tumors. Even within cervical cancers, which HPV-18 is generally accepted as causing^13^, HPV-18 showed 69% lower average oncogene expression than did HPV-16. Considering all samples with positive expression of at least one HPV type, the difference of 2122 mean reads for HPV-18 versus 6893 mean reads for HPV-16 showed a significantly lower expression level for HPV-18 than HPV-16 (Fig. 2, sign test, p < 10^−18^).

## Discussion

Here we provide evidence of non-zero prevalence of HPV in ovarian cancers in North America. In 1.5% of ovarian tumors, we found transcripts of the oncogenes of HPV-18, but not HPV-16. Our detection in ovarian cancer of only HPV-18 contrasted with results of past DNA detection studies, in which the most common type of HPV in ovarian cancers was HPV-16, independent of geographical region^3^, and also with a study of 56 ovarian cancers in Hong Kong, which found that relative to HPV-16, DNA of HPV-18 was, “extremely rare”^14^. Our observation in ovarian tumors of HPV-18 and not HPV-16 also differed from our results on cervical tumors, which were positive more often for type 16 than 18. Higher prevalence of HPV-16 than HPV-18 has also been reported for oropharyngeal cancers^15^. However, although HPV-16 is the most prevalent type in human genital neoplasias in general, HPV-18 does tends to dominate in glandular carcinomas such as those we studied here^13^.

Knowing that a subtype of ovarian cancers expresses a well-characterized non-human gene might help in designing targeted therapies for this sub-type. More importantly, this subtype may be largely preventable. Results of clinical trials in other gynecological cancers suggest that the incidence of HPV-18 infection and resultant cancers, “will decrease at several anatomical sites in women who receive the prophylactic HPV vaccines before exposure to the virus”^16^.

Our results implicate HPV as being involved with some cases of ovarian cancer, but do not establish causality. Medical research has long grappled with criteria for determining whether a microorganism causes a disease, and these criteria have been revisited for the modern age of sequence-based identification of microbial pathogens^17^. Under these updated criteria, HPV is a candidate for causing some cases of ovarian cancer because viral oncogene expression is documented in clinical cancer samples, and because this makes biological sense as a cause. Viral oncogenesis would be consistent with the known exposure of ovarian epithelium to HPV virus, and with the known mechanisms of viral carcinogenesis. However, we could not test one key criterion for sequence-based attribution of causation, which is that, “fewer, or no, copy numbers of pathogen-associated nucleic acid sequences should occur in hosts or tissues without disease”^17^.

It seems plausible that the viral oncogene expression we found plays an active role in ovarian oncogenesis, rather than being present only incidentally as part of systemic infection. These are known oncogenes that clearly cause epithelial malignancies in other sites that HPV infects. A genomic fragment of HPV-18 containing the *E6* and *E7* genes is sufficient for keratinocyte cell transformation *in vitro*^18^. In epithelial cells of other organs, the HPV E6 oncoprotein targets p53 protein for degradation, activates telomerase expression, and modulates the activities of PDZ domain-containing proteins and tumor necrosis factor receptors^19^. It seems unlikely that these molecular mechanisms of transformation operate in the infected epithelial cells of some organs and not others, but their role in ovarian epithelium specifically could be clarified by comparing viral oncogene expression in cancer samples versus adjacent normal tissues. Associated normal samples were reportedly included in the clinical sampling protocol for TCGA^6^, but were not subject to RNA-seq analysis. Such a comparison with ovarian cancer transcripts would be a useful follow-up to the study reported here.

Our results raise the question of why some previous studies failed to detect HPV DNA in ovarian tumors from North America. In some cases the answer may involve a combination of looking for the wrong HPV types, and using low-sensitivity assays. For example, one study^20^ concluded that “there appears to be no association between HPV and ovarian neoplasia”, after testing for low-risk types using high-sensitivity PCR, but testing for high-risk types using only high-stringency (low-sensitivity) Southern hybridization. In several other studies though, PCR amplification has also failed to detect any HPV DNA in ovarian tumors from North America. Here the problem clearly was sample size that was inadequate to detect the drastically lower prevalence in ovarian versus cervical cancer. Although a recent meta-analysis of prevalence^3^ concluded that “it seems unlikely that HPV plays a role in ovarian cancer among Western European and American women”, the American studies cited were very small, with at most 20 cases of epithelial ovarian cancer. If the true prevalence of HPV in ovarian epithelial cancers is 1.5%, as we estimate, then under the binomial distribution, even with a perfectly accurate assay, the probability of successfully detecting any HPV among 20 cases is only 26%. Even pooling all five of the North American studies cited^3^ provides only 88 cases of ovarian cancer, which, with a perfect assay, would still have had a 26% probability of failing to detect any HPV in a population with an actual prevalence of 1.5%.

Two previous studies examined TCGA data for expression of HPV oncogenes in cancers including ovarian. Neither of these studies detected HPV-18 in ovarian cancer^21^,^22^. The reason for this discrepancy with our study seems to be different criteria for the number of aligned reads required to report a sample as being HPV-positive. It is important to note that none of these criteria, including ours, has been empirically validated. In light of new technology that makes sequencing and the alignment of sequences against large references ever more accessible, this raises important questions about appropriate sequence alignment cutoffs and the need for their biological validation.

Given that the above-mentioned studies^21^,^22^ used criteria for the number of aligned reads that were several orders of magnitude higher than ours, some justification may be required for why we considered our smaller number of aligned reads per sample to be valid evidence of oncogene expression, rather than false positives. Firstly, our quality filtering of cDNA reads reduced the possibility of false positives due to sequencing errors. Secondly, five of the six ovarian cancer samples positive for HPV-18 had alignments indicating expression of multiple HPV-18 genes.

Finally, much of our confidence in our oncogene alignments stems from the fact that the paired-end sequencing reads that aligned to *E6* or *E7* have a length of 75 base pairs each and 100% identity to the HPV-18 oncogenes only. A long alignment such as that provided by 75 base-pair reads is robust and does not require high read depth to be considered valid. Regarding the oncogenes specifically, most of the positive samples had concordant alignments, meaning that the paired mates of the paired-end sequencing reads both aligned to the HPV-18 oncogene with the expected orientation and distance, indicating a match over the entire length of the RNA molecule of which the 75 base-pair ends were sequenced. It is extremely unlikely that a series of sequencing errors could have generated such alignments. For all these reasons, we believe our results demonstrate that claims of no involvement of HPV in ovarian cancer should not be viewed as conclusive.

Our bioinformatics approach not only revealed HPV-18 oncogene expression in ovarian tumorigenesis, but also should motivate the continued use of TCGA data for studies of cancer genes of viral as well as human origin.

## Conclusions

Contrary to the most recent review of this topic^3^, our results do show that HPV is present and active in some cases of ovarian cancer in North America, including expression of specific oncogenes shown to be sufficient for in vitro transformation. In contrast to cervical cancer, the only HPV type actively expressed in ovarian malignancies was HPV-18, not HPV-16. In searching for HPV in ovarian cancer, it may be important to test for HPV-18 instead of HPV-16, and to recognize that this virus typically exhibits lower gene expression.

As of 2014, the number of US ovarian cancer cases annually is estimated at 21,980, with 14,270 deaths annually^2^. Because clinical trials suggest that HPV-18 infection and resultant gynecological cancers can be reduced by available vaccines, those vaccines may have potential for reducing ovarian cancer. If this succeeded, reducing ovarian cancer cases by the 1.5% involving preventable viral infection would mean 330 fewer cases and 214 fewer deaths per year.

## Methods

We analyzed tumor RNA transcriptomes for expression of genes of 14 different HPV types. We focused on two well-studied oncogenes of HPV, genes *E6* and *E7*. These are often used in assays for HPV virus^23^,^24^,^25^ and are required for cell transformation in cervical cancer^7^,^8^. Moreover, E6 and E7 transcripts of HPV are found in virtually all HPV-positive cervical neoplasia specimens^8^. It has also been reported that unlike some other parts of the HPV genome, the region containing *E6* and *E7* is never lost during integration of viral DNA into host genomic DNA^23^. We used data from The Cancer Genome Atlas (TCGA, https://tcga-data.nci.nih.gov). The data were cDNA sequences from primary malignant tumors, generated by Illumina RNA-seq. The ovarian tumors included in this data set were classified as serous cystadenocarcinoma. From the NCI database at https://tcga-data.nci.nih.gov, we used the code in the tissue ID field (“TSS_ID”), to determine what institution had provided the tissue sample. In order to limit our study to North America, we excluded all samples from other regions. The North American sites where ovarian tumor samples were collected are listed in Table 3.

For validation of our methods, we used cervical tumors as a positive control for HPV detection (a high prevalence was expected here), and glioblastoma tumors as a negative control (zero prevalence was expected here). In total, we examined cDNA data from 251 cervical tumors, 156 glioblastoma tumors, and 405 ovarian tumors. Each sample came from a different patient. The data as originally downloaded contained duplicate entries for many ovarian samples, but these were omitted from all analyses.

The cDNA sequences from each tumor sample were downloaded from CGHub (https://cghub.ucsc.edu). This database is updated on an ongoing basis, but we used all available sequence data as of Sept. 1, 2014. The downloads were BAM files, with notations of alignment to the human genome. Because we were interested in non-human (viral) genes, we used only the reads that did not map to the human genome.

To convert the BAM files into fastq files containing only the unmapped reads, we used the program bam2fastq, with the following command: bam2fastq --no-aligned --no-filtered -o unmapped.fastq.

Before analyses, to filter sequences for quality, we excluded any paired-end read sequence where the sequence for either paired read had more than 3 bases that did not have a phred quality score^26^ of at least 15, following the PathSeq protocol^27^. We then used Bowtie 2 software^28^ to determine whether the remaining non-human reads would align to HPV oncogene reference sequences. All of the reference sequences we used are documented at the HPV sequence database PaVE (http://pave.niaid.nih.gov), maintained by the National Institute of Allergy and Infectious Diseases. Each reference sequence is available from the National Center for Biotechnology Information at http://www.ncbi.nlm.nih.gov.

We created a Bowtie 2 index from all the HPV types considered and then used the following Bowtie 2 command for alignment, where "HPVreferences" is the basename of the Bowtie 2 index we created: bowtie2 -x HPVreferences -1 1_unmapped.fastq -2 2_unmapped.fastq.

For each tumor sample, we digitally searched for oncogene transcripts of HPV types 6, 11, 16, 18, 31, 33, 35, 39, 45, 51, 52, 56, 58, and 59. All of these are considered high-risk types except for types 6 and 11^29^. For each Bowtie 2 analysis, we recorded the number of reads from that tumor sample that aligned to each tested HPV oncogene reference sequence. We designated a sample as positive for a given HPV type if the sample produced at least one read that aligned to the corresponding HPV reference sequence. As a proxy for the amount of HPV oncogene RNA in a sample, we also recorded the total number of reads aligning to the HPV reference sequence^30^.

Our methods were carried out in accordance with relevant guidelines and regulations. Our research protocol and our access to TCGA data was approved by the TCGA Data Access Committee. All original specimens were obtained from patients with appropriate informed consent, and with approval from the multiple relevant institutional review boards at the fourteen institutions that dealt with the human subjects^6^, which are listed in Table 3.

## Author Contributions

This study was conceived and designed by J.W.P. and R.M.F. Data analysis was carried out by P.A.O. and P.R. Interpretation was performed by R.M.F., J.W.P., P.A.O. and P.R. The manuscript was written by J.W.P., P.A.O., P.R. and R.M.F. The project was supervised by J.W.P. and R.M.F.

## Figures and Tables

**Table 1:** Prevalence of HPV oncogene transcripts in cervical, glioblastoma, and ovarian tumors

Tumor type	No. of Positive Samples	Total No. of Samples	Percent of Samples Positive for HPV mRNA
Cervical	245	251	97.6%
Glioblastoma	0	156	0%
Ovarian	6	405	1.5%

**Table 2:** Number of cervical tumor samples testing positive for HPV by HPV type (of 251 samples tested for 14 types). Note that some samples were positive for more than one type, so that rows sum to more than 100% of total

	HPV Type													
	6	11	16	18	31	33	35	39	45	51	52	56	58	59
# of samples positive	**0**	**0**	**175**	**52**	**12**	**19**	**17**	**9**	**19**	**10**	**10**	**2**	**9**	**4**
% of positive samples	0%	0%	70%	21%	5%	8%	7%	4%	12%	4%	4%	1%	4%	2%

**Table 3:** Origins of ovarian tumor samples studied, with results. All data are from the TCGA database. Note that sample sizes are too small for precise prevalence estimates by province or state. Many of those apparent differences may reflect random sampling error

Source Institution	Province or State	# of cases	# positive cases	% positive
British Columbia Cancer Agency	British Columbia	11	1	9.1%
Gynecologic Oncology Group	California	24	2	8.3%
Duke University Medical Center	North Carolina	35	2	5.7%
University of Pittsburgh	Pennsylvania	41	0	0.0%
Mayo Clinic - Rochester	Minnesota	42	1	2.4%
Roswell Park Cancer Institute	New York	7	0	0.0%
Fox Chase Cancer Center	Pennsylvania	8	0	0.0%
Washington University School of Medicine	Missouri	90	0	0.0%
MD Anderson Cancer Center	Texas	9	0	0.0%
Harvard Medical School	Massachusetts	10	0	0.0%
Cedars-Sinai Medical Center	California	27	0	0.0%
UC San Francisco	California	22	0	0.0%
Memorial Sloan-Kettering Cancer center	New York	72	0	0.0%
International Genomics Consortium	Arizona	7	0	0.0%
Total	all sites	405	6	1.5%
